# Fur-type transcriptional repressors and metal homeostasis in the cyanobacterium *Synechococcus* sp. PCC 7002

**DOI:** 10.3389/fmicb.2015.01217

**Published:** 2015-10-31

**Authors:** Marcus Ludwig, Tiing Tiing Chua, Chyue Yie Chew, Donald A. Bryant

**Affiliations:** ^1^Department of Biochemistry and Molecular Biology, The Pennsylvania State University, University ParkPA, USA; ^2^Department of Chemistry and Biochemistry, Montana State University, BozemanMT, USA

**Keywords:** iron homeostasis, zinc homeostasis, Zur, Per, SmtB, ArsR, transcriptome profiling, photosynthesis

## Abstract

Metal homeostasis is a crucial cellular function for nearly all organisms. Some heavy metals (e.g., Fe, Zn, Co, Mo) are essential because they serve as cofactors for enzymes or metalloproteins, and chlorophototrophs such as cyanobacteria have an especially high demand for iron. At excessive levels, however, metals become toxic to cyanobacteria. Therefore, a tight control mechanism is essential for metal homeostasis. Metal homeostasis in microorganisms comprises two elements: metal acquisition from the environment and detoxification or excretion of excess metal ions. Different families of metal-sensing regulators exist in cyanobacteria and each addresses a more or less specific set of target genes. In this study the regulons of three Fur-type and two ArsR-SmtB-type regulators were investigated in a comparative approach in the cyanobacterium *Synechococcus* sp. PCC 7002. One Fur-type regulator controls genes for iron acquisition (Fur); one controls genes for zinc acquisition (Zur); and the third controls two genes involved in oxidative stress (Per). Compared to other well-investigated cyanobacterial strains, however, the set of target genes for each regulator is relatively small. Target genes for the two ArsR-SmtB transcriptional repressors (SmtB (SYNPCC7002_A2564) and SYNPCC7002_A0590) are involved in zinc homeostasis in addition to Zur. Their target genes, however, are less specific for zinc and point to roles in a broader heavy metal detoxification response.

## Introduction

Metal uptake and homeostasis are important functions for most bacteria and are very important for chlorophototrophs because of the extensive use of metals in photosynthetic complexes. Iron availability is a key issue for marine microorganisms because iron availability limits microbial growth and productivity in marine environments (Coale et al., 1996; Morel, 2008; Behrenfeld and Milligan, 2013). Fur-type transcriptional regulators are widespread among prokaryotes and are involved in regulating the uptake and homeostasis of several metal ions and related functions (e.g., oxidative stress response; Lee and Helmann, 2007; Fillat, 2014; O’Brian, 2015; Porcheron and Dozois, 2015). Control of iron uptake was the first function to be described for such a regulator, and subsequently the protein was named ferric uptake regulator (Fur) (Hantke, 1981; Bagg and Neilands, 1987a,b). Fur-type regulators are homodimers with both DNA and metal-binding domains (Bagg and Neilands, 1987a). These regulators have commonly been described as transcriptional repressors that bind to the DNA operator sites when loaded with co-repressor (Lee and Helmann, 2007; Osman and Cavet, 2010), but there are also examples in which Fur-type regulators act as transcriptional activators (Delany et al., 2001). Indirect activation of gene expression can also occur via small RNAs: the Fur-type regulator acts as a transcriptional repressor for a sRNA, and as long as the sRNA level is low, the target genes are expressed, whereas de-repression of the sRNA results in a degradation of target RNAs (Massé and Gottesman, 2002). Small RNAs have been identified to be involved in regulation of iron homeostasis in the cyanobacterium *Synechocystis* sp. PCC 6803 (Hernández-Prieto et al., 2012). Besides iron homeostasis, Fur-type regulators have been described for zinc, manganese and nickel homeostasis; have been described as regulators for oxidative stress responses; and play direct or indirect roles in several other cellular functions (Lee and Helmann, 2007; Fillat, 2014). The amino acid sequences of these regulators are highly similar, and subtle changes can alter the specificity of metal binding. For example, it has been demonstrated that an exchange of a single amino acid transformed an iron-responsive Fur-type regulator into a protein as sensitive to H_2_O_2_ as a natural, peroxide-sensing Fur-type regulator (Parent et al., 2013). Besides the Fur-type transcriptional regulators there are transcriptional repressors of the ArsR-SmtB family that are also involved in regulating metal homeostasis. ArsR-SmtB family repressors respond to high levels of metal ions that are stress-inducing or toxic for the cell; metal ions bind to the regulator as a de-repressor for this repressor family (Busenlehner et al., 2003; Osman and Cavet, 2010).

*Synechococcus* sp. strain PCC 7002 (hereafter *Synechococcus* 7002) is a euryhaline, unicellular cyanobacterium that is capable of growth over a wide range of NaCl concentrations and is extremely tolerant of high light intensities (Batterton and Van Baalen, 1971; Nomura et al., 2006; Bernstein et al., 2014). It is a very fast growing and well-characterized model organism that is naturally transformable, and convenient methods for the heterologous expression of genes have been developed (Stevens and Porter, 1980; Xu et al., 2011). Cyanobacteria have been shown to possess different Fur-type transcriptional regulators, and studies in various cyanobacterial strains have assigned functions and regulation patterns to single Fur-type regulators (Ghassemian and Straus, 1996; Kobayashi et al., 2004; Hernández et al., 2006; González et al., 2010; Napolitano et al., 2012). The genome of *Synechococcus* 7002 encodes three genes that are annotated as Fur-type regulators. In this study we performed deletion mutagenesis and, on the basis of transcriptome profiling, we assigned functions to these three transcriptional regulators of this cyanobacterium. In addition to the *fur* genes of *Synechococcus* 7002, we investigated the regulons of two transcriptional repressors of the ArsR-SmtB family that are involved in responses to heavy metal stress and toxicity.

## Materials and Methods

### Bacterial Strains and Culture Conditions

Wild-type and mutant strains of *Synechococcus* sp. PCC 7002 were grown in liquid culture and on 1.5% (w/v) agar plates in medium A supplemented with 1 mg NaNO_3_ ml^-1^ (designated as medium A^+^) as previously described (Stevens and Porter, 1980; Ludwig and Bryant, 2011). Liquid cultures were grown in tubes containing medium (25 mL) at 38°C with continuous irradiation with 250 μmol photons m^-2^ s^-1^, and the cultures were sparged with 1% (v/v) CO_2_ in air. The following antibiotic concentrations were added to the medium whenever appropriate: spectinomycin, 50 μg mL^-1^; erythromycin, 20 μg mL^-1^. Unless otherwise stated, cultures for growth rate experiments were grown without antibiotics, whereas cultures for RNA analyses were grown in the presence of the respective antibiotics. Cell growth was monitored by measuring the optical density at 730 nm (OD_730 nm_; OD_730 nm_ = 1.0 is equivalent to 1.0 ± 0.2 × 10^8^ cells mL^-1^) with a Genesys 10 spectrophotometer (ThermoSpectronic, Rochester, NY, USA). Cultures for RNA analyses were inoculated at an OD_730 nm_ between 0.05 and 0.1 from pre-cultures that had been grown under the same conditions. When these cultures reached an OD_730 nm_ = 0.7, three independently grown, replicate cultures were pooled. Cells derived from 25 mL aliquots of the cultures were rapidly centrifuged (5 min, 5000 × *g*, 4°C), and the cell pellets were rapidly frozen in liquid nitrogen and stored at -80°C until required.

Fe-limitation was induced by adding the Fe-binding chelator, deferoxamine mesylate B, to the culture as previously described (Ludwig and Bryant, 2012a). At OD_730 nm_ = 0.35 deferoxamine mesylate B (720 μM final concentration) was added to a culture in A^+^ medium (containing 14.4 μM iron). The resulting culture was grown under standard conditions until OD_730 nm_ = 0.7.

Medium A^+^ contains 2.3 μM Zn^2+^, and to induce zinc stress, aliquots of a ZnCl_2_ stock solution (100 mM ZnCl_2_, buffered in 1 M Tris at pH 8.0) were added to produce final Zn^2+^ concentrations of 12.3 μM, 22.3 μM, 52.3 μM, 102 μM, and 202 μM. Cultures subjected to Zn-limitation were grown in modified A^+^ medium without added ZnCl_2_ (ZnCl_2_ was omitted from the trace element solution). After several transfers on “Zn-free” A^+^ medium (to dilute any residual Zn^2+^), the heavy metal chelator TPEN was added at a final concentration of 50 μM to lower the availability of divalent cations (Anderegg et al., 1977). TPEN was prepared as a 50 mM stock solution in dimethylsulfoxide.

### Construction of Knock-out And Knock-down Strains

For inactivation of SynPCC7002_A1649 (*fur*), SynPCC7002_A1836 (*per*), SynPCC7002_A2498 (*zur*), SynPCC7002_A0590, and SynPCC7002_A2564 (*smtB*), ∼1000-bp regions upstream and downstream of the respective genes were amplified by PCR and ligated to drug marker cassettes. Primers #1 and #2 were used to amplify the upstream region and #3 and #4 for amplification of the downstream region of SynPCC7002_A1649 (see **Table 1** for oligonucleotide sequences). Primers #5 and #6 (upstream region) and primers #7 and #8 (downstream region) were used for amplification of SYNPCC7002_A1836 flanking regions; and primers #9 and #10 (upstream region) and primers #11 and #12 (downstream region) were used to amplify the flanking regions of SYNPCC7002_A2498, respectively. Oligonucleotides #13 and #14 and oligonucleotides #15 and #16 were used for amplification of the SYNPCC7002_A0590 upstream and downstream flanking regions; and for amplification of SYNPCC7002_A2564 flanking regions primers #17 and #18 (upstream fragment) and #19 and #20 (downstream fragment) were used. The PCR fragments were digested using the enzymes listed in **Table 1**. The *aadA* gene, conferring streptomycin and spectinomycin resistance, was excised as a 1040-bp Eco53kI/HindIII fragment from plasmid pSRA81 (Frigaard et al., 2004b) or as a 1091-bp Eco53kI fragment from plasmid pSRA2 (Frigaard et al., 2004a). The *ermC* gene, conferring erythromycin resistance, was excised from pRL409 (Elhai and Wolk, 1988) as a 1503-bp EcoRV-HindIII fragment. The respective fragments were mixed at a 3:1:3 ratio of the upstream flank to the antibiotic resistance cassette to the downstream flanking region and ligated with T4 DNA ligase. The ligation reactions were directly used to transform *Synechococcus* 7002 as described previously (Frigaard et al., 2004b).

**Table 1:** Oligonucleotide primers used in this study.

Number	Sequence (relevant restriction sites underlined, 5′ to 3′)
#1	AAATCTAGAGCAATGGTGCTAATCTCCTC (XbaI site underlined)
#2	TTTGATATCATATTTTTCAACAAGTTCAG (EcoRV site underlined)
#3	AAAAAGCTTAATCGAGGCGATCGCATTTGACGGTAAC (HindIII site underlined)
#4	TTTCTCGAGGCACTCCGCAGGGCTAAATC (XhoI site underlined)
#5	AAATCTAGACCGCAATCTGGGTGCCGAGG (XbaI site underlined)
#6	TTTGATATCAAGGCTTGTTCGTTGCAAAATC (EcoRV site underlined)
#7	AAAGATATCTAACTTGTTTAAATAATCTG (EcoRV site underlined)
#8	TTTCTCGAGGGGTATCGCTTTGGGCACAG (XhoI site underlined)
#9	AAATCTAGATGATGGCGGGAATCCTCGTC (XbaI site underlined)
#10	TTTGATATCATTAGGCTTCTGGTTGGGTG (EcoRV site underlined)
#11	ACCCAAGCTTCAGGCCGATAATGCTGAATG (HindIII site underlined)
#12	TTTCTCGAGATATAGGAGTGGCCATCCTC (XhoI site underlined)
#13	AAATCTAGAACTGCTCGCCGCCGTGGAACAG (XbaI site underlined)
#14	CCCCCCGGGCATTAAATCAAAAATAATTGATGC (SmaI site underlined)
#15	AAAAAGCTTAAAACCCCAACCCAGAATATAAATC (HindIII site underlined)
#16	CCCCTCGAGAGCTGCATCTTCATAGGTAAATCCC (XhoI site underlined)
#17	TGCTCTAGACAAGGGCAATATGAACTGGC (XbaI site underlined)
#18	TCCGATATCTTGGCATCCCCCAAGACACC (EcoRV site underlined)
#19	CCCAAGCTTACACACCCACTAATCAACGC (HindIII site underlined)
#20	CCGCTCGAGAAGGTTACGAAGTTCATGGC (XhoI site underlined)

### RNA Preparation, RNA Sequencing, and Data Analysis

RNA samples for cDNA library construction were prepared from frozen cell pellets derived from 25-ml aliquots of liquid culture (see above). The RNA preparation and quantitation was performed as described previously (Ludwig and Bryant, 2011), and construction of cDNA libraries and SOLiD^TM^ sequencing was performed in the Genomics Core Facility at The Pennsylvania State University (University Park, PA). The cDNA libraries were constructed using SOLiD^TM^ Whole Transcriptome Analysis Kit (Applied Biosystems) and were barcoded by using the SOLiD^TM^ Transcriptome Multiplexing Kit (Applied Biosystems). SOLiD^TM^ ePCR Kit and SOLiD^TM^ Bead Enrichment Kit (both Applied Biosystems) were used for processing the samples for sequencing, and the SOLiD^TM^ 4 or SOLiD^TM^ 5500 protocol (Applied Biosystems) was used for sequencing. The sequence data have been submitted to the NCBI Sequence Read Archive (SRA) under accession numbers SRR833548, SRR1057998–SRR1058000, SRR2179721–SRR2179730.

The cDNA sequences were mapped using the Burrows–Wheeler algorithm (Li and Durbin, 2009). The *Synechococcus* 7002 genome and the sequences for the antibiotic resistance cassettes served as the reference set; four mismatches within the 50-bp reads were allowed (>90% sequence identity), and sequences mapping to non-unique locations were excluded. Further processing, analysis and comparisons of data sets were performed as described previously (Ludwig and Bryant, 2011). Statistical analysis was performed using the chi-square test if one data set was available for each condition or the *z*-test for comparisons to the WT standard sample, for which three independent data sets were available. The data for all protein-coding open reading frames (ORFs) derived from these analyses are listed in the Supplementary Tables S1 and S2.

### Measurement of Reactive Oxygen (ROS) and Nitrogen Species (RNS)

The cytosolic ROS/RNS contents in cells was probed using the membrane-permeant indicator CM-H_2_DCFDA from Molecular Probes, Invitrogen (Eugene, OR), which reacts with ROS and RNS and is detected by fluorescence emission. The protocol recommended by the manufacturer was used. Cultures of WT and mutant strains were grown to OD_730 nm_ = 0.7 under standard conditions, then CM-H_2_DCFDA was added at a final concentration of 5.0 μM, and the cultures were incubated under high irradiance (830 μmol photons m^-2^ s^-1^ (at 38°C and standard sparging conditions). For the subsequent fluorescence measurements the OD_730 nm_ was adjusted to 0.5 for all samples and the fluorescence was measured using an SLM8000-based spectrofluorometer modernized for computerized, solid-state operation by On-Line Instrument Systems Inc., (Bogart, GA). The excitation wavelength was 493 nm, and the emission spectrum was recorded from 510 to 610 nm; the amplitude at 523 nm was used for calculations and comparisons (Zhu et al., 2010).

## Results

### Deletion of Genes Encoding Fur-type Regulators

**FIGURE 1:**
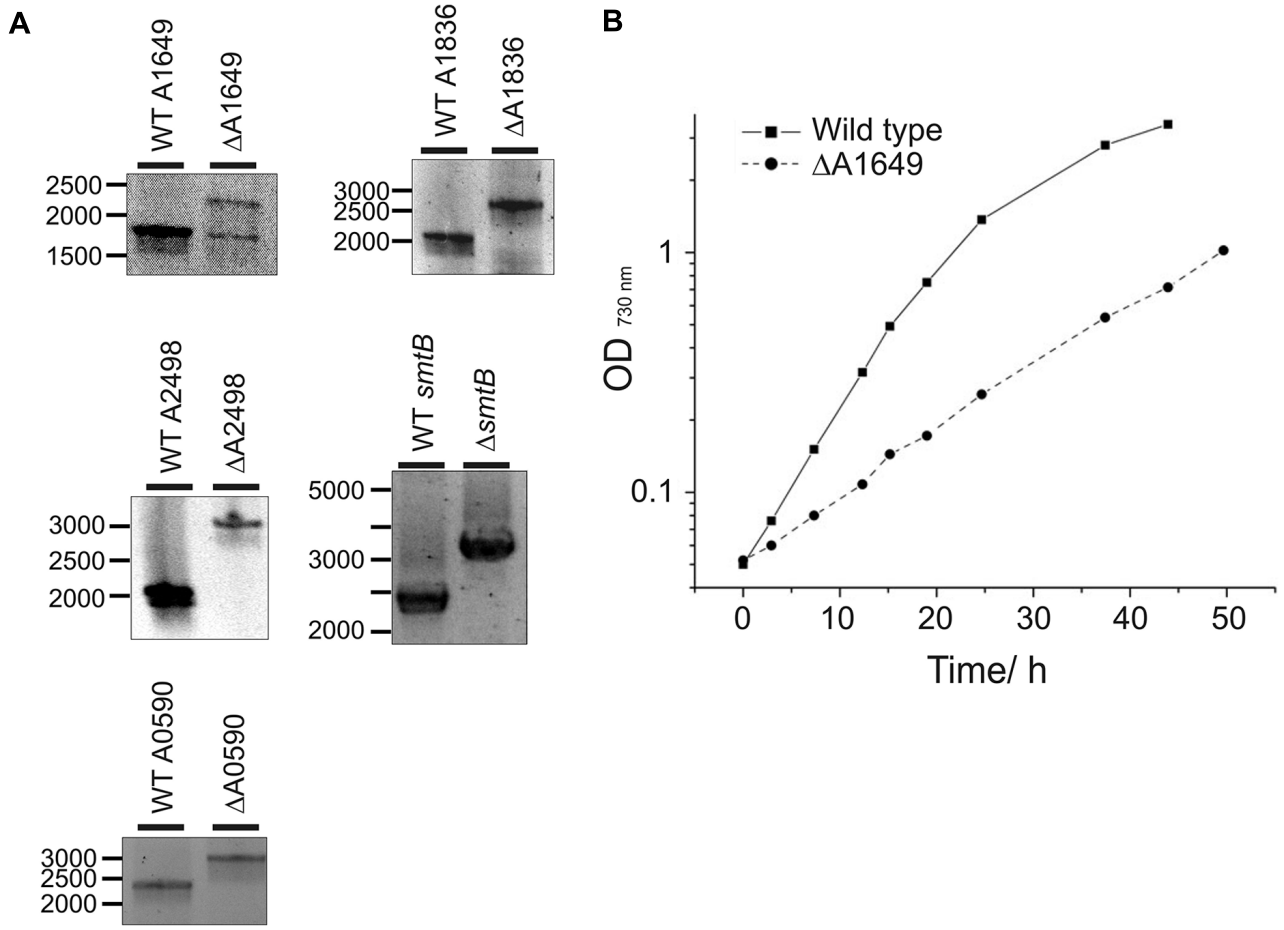
**Segregation analysis of deletion mutants and growth of the merodiploid SYNPCC7002_A1649 strain. (A)** Electrophoretic analysis of PCR amplicons produced using DNA templates derived from the wild-type (WT) strain and SYNPCC7002_A1649 (ΔA1649), SYNPCC7002_A1836 (ΔA1836), SYNPCC7002_A2498 (ΔA2498), SYNPCC7002_A2564 (*ΔsmtB*), and SYNPCC7002_A0590 (ΔA0590) mutant strains of *Synechococcus* 7002. For the SYNPCC7002_A1649 (*fur*) mutant, the expected amplicon size was 1.82 kb for WT and 2.35 kb for the mutant, respectively. Note that the mutant is a stable merodiploid with approximately equal copies of each allele (*fur* and *fur::aadA*). For SYNPCC7002_A1836 (*perR*), the expected amplicon size was 2.12 kb for WT and 2.72 kb for the mutant. For SYNPCC7002_A2498 (*zur*), the expected amplicon size was 1.99 kb for WT and 3.11 kb for the deletion mutant, respectively. The amplicon sizes for *smtB* were 2.33 kb (WT) and 3.14 kb (mutant), and for SYNPCC7002_A0590 amplicons had 2.29 kb (WT) and 3.02 kb (mutant), respectively. **(B)** Growth of *Synechococcus* 7002 WT and the merodiploid SYNPCC7002_A1649 (*fur*)::*aadA* strain in A^+^ medium. The growth medium for the latter strain contained 50 μg/mL spectinomycin for continuous selection. The cultures were grown under standard growth conditions, and representative growth curves for these two strains are displayed. Growth of the other mutant strains investigated in this study was wild type-like.

The *Synechococcus* 7002 genome encodes three members of the helix-turn-helix family of Fur-type metalloregulators. One gene was originally annotated as the *fur* gene based on sequence homology (locus tag number SYNPCC7002_A1649) (pfam 01475), whereas the other two paralogs (locus tag numbers SYNPCC7002_A1836 and SYNPCC7002_A2498) were not specifically assigned functions during annotation. To explore the function of these three transcriptional regulators, deletion mutants were constructed for all three *fur* homologs as described in the Section “Materials and Methods”. Wild-type and mutant alleles in transformants for the deletion of the SYNPCC7002_A1836 and SYNPCC7002_A2498 genes segregated fully, which demonstrates that the products of these genes are not essential for growth under standard conditions. However, PCR analysis of the WT and mutant alleles in transformants for the deletion of SYNPCC7002_A1649 showed a ∼1:1 ratio of WT and mutant amplicons that was stable over numerous transfers and varying light conditions; the agar plates used for selection were incubated under low irradiance levels (**Figure 1A**). This result indicates that SYNPCC7002_A1649 is essential for growth at least under the conditions that were tested in this study.

Strains in which SYNPCC7002_A1836 or the SYNPCC7002_A2498 were deleted grew identically to the WT under standard temperature and light conditions in A^+^ growth medium (data not shown). However, the growth of the merodiploid SYNPCC7002_A1649 strain was severely impaired compared to the WT and all other mutant strains (**Figure 1B**). Although antibiotics were not necessary for strains deleted for SYNPCC7002_A1836 and SYNPCC7002_A2498, antibiotics had to be added to the culture medium for the SYNPCC7002_A1649 merodiploid strain to maintain selection on the drug marker that was introduced to delete the SYNPCC7002_A1649 gene. Because streptomycin was present during growth of this strain, and because the target gene was only partially deleted, calculation of a valid growth rate is not possible for this particular strain. However, in general we have found that the *aadA* gene and streptomycin have at most a very modest effect on the growth of mutants in *Synechococcus* 7002.

### Transcriptome Profiling of Mutant Strains for the Fur Family

**FIGURE 2:**
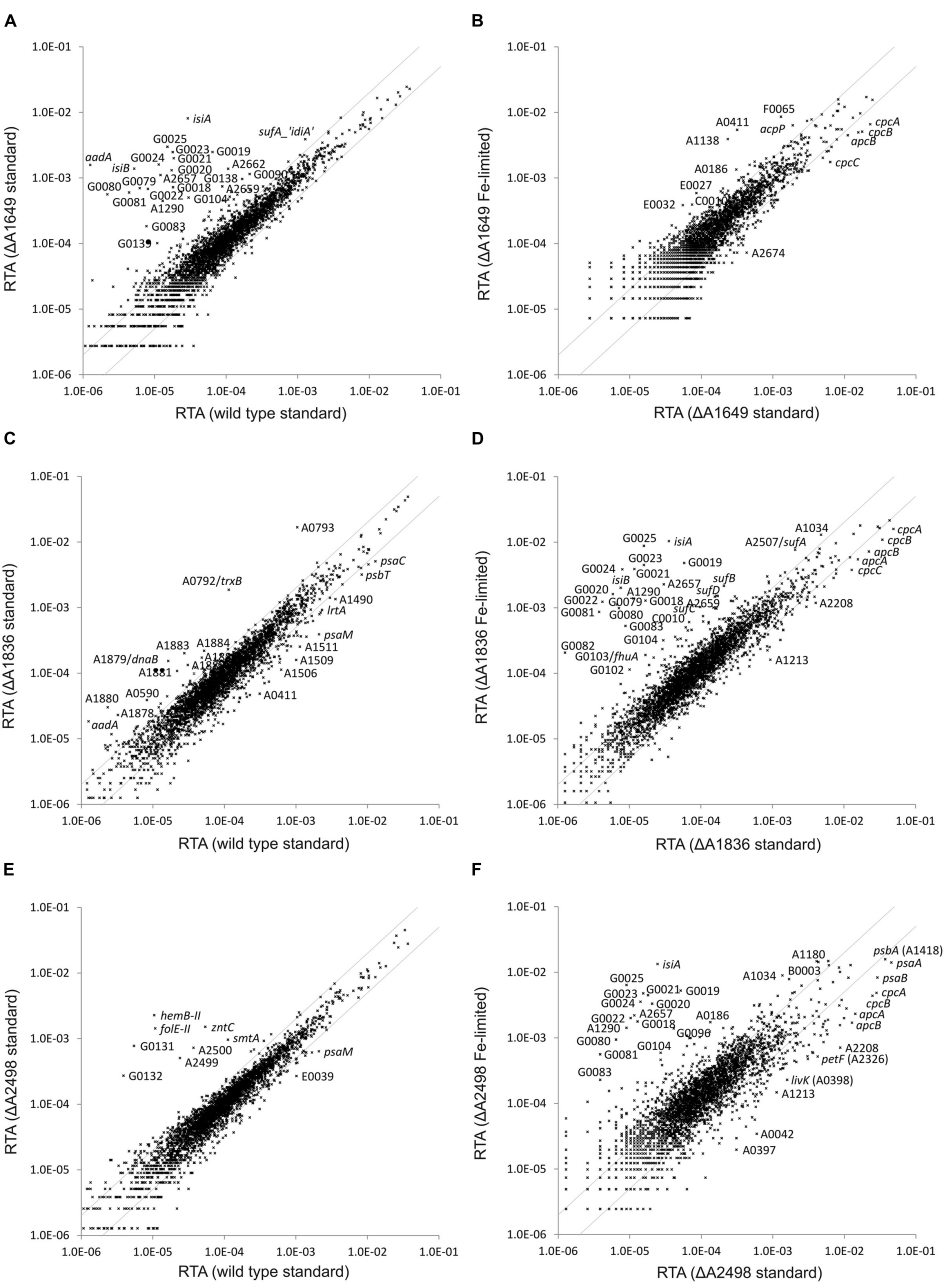
**Relative transcript abundances (RTAs) for the SYNPCC7002_A1649 (*fur*), SYNPCC7002_A1836 (*perR*) and SYNPCC7002_A2498 (*zur*) mutant strains compared to WT *Synechococcus* 7002 and of the mutant strains subjected to iron limitation compared to standard growth conditions.** The scatter plots show **(A)** the RTAs of transcripts from the merodiploid SYNPCC7002_A1649 (*fur*) strain compared to the wild type when both strains were grown under standard conditions; **(B)** RTAs of the merodiploid SYNPCC7002_A1649 strain subjected to iron limitation compared to standard growth conditions; **(C)** the RTAs of the SYNPCC7002_A1836 (*perR*) deletion mutant compared to the wild type when both were grown under standard conditions; **(D)** the RTAs of SYNPCC7002_A1836 deletion mutant subjected to iron limitation compared to those for standard growth conditions; **(E)** the RTAs of the SYNPCC7002_A2498 (*zur*) deletion mutant strain compared to a wild type culture, both grown under standard conditions; and **(F)** the RTAs of an iron-limited culture of the SYNPCC7002_A2498 deletion mutant compared to a culture grown under standard conditions. The values for the wild type under standard conditions were calculated as the mean of three independent biological replicates. The gray lines show twofold changes in either direction. Selected genes are identified by gene locus or abbreviated locus tag number (e.g., G0025 = SynPCC7002_G0025). For further details, see text.

Global transcriptome profiling of the regulator mutants was performed in order to identify target genes and their functions in the regulons for each of the three Fur-type regulators. The transcriptome of the merodiploid SYNPCC7002_A1649 strain was compared to that obtained for the WT when both strains were cultivated under standard growth conditions (**Figure 2A**). Many genes showed much higher transcript levels in the mutant strain compared to the WT (see Supplementary Tables S2 and S3). Those genes encode proteins and enzymes involved in iron acquisition (siderophore biosynthesis, components of different types of Fe-transporters, etc.) or encode alternative proteins that replace proteins containing Fe-S clusters [e.g., *isiB* (flavodoxin)] (Leonhardt and Straus, 1992). Transcript levels for all of these genes have been reported to increase under iron limitation in *Synechococcus* 7002 (Ludwig and Bryant, 2012a), and in fact the comparison of mutant to WT under standard conditions closely resembles the comparison of an iron-limited WT profile to the WT profile under standard growth conditions (Ludwig and Bryant, 2012a). Transcript levels resulting from the SYNPCC7002_A1649 gene were about 2.5-fold lower in the merodiploid strain compared to the WT, which approximately reflects the 1:1 ratio of mutant and WT gene that was detected in the PCR analysis (**Figure 1A**). Fe-limitation of the merodiploid SYNPCC7002_A1649 strain had almost no effect on the global transcriptome of that strain when compared to standard growth conditions (**Figure 2B**). Only very few genes encoding enzymes/proteins related to iron uptake or metabolism showed further induction under Fe-limitation of a culture of the merodiploid SYNPCC7002_A1649 strain. Genes that have been previously reported to have decreased mRNA levels upon limitation of several nutrients (e.g., nitrogen, sulfur, phosphorous, and carbon) (Ludwig and Bryant, 2012a) displayed lower transcript levels in the SYNPCC7002_A1649 merodiploid strain upon Fe-limitation (e.g., *cpcA, cpcB, cpcC, apcB*, etc.; see Supplementary Table S3).

The global transcriptome of the SYNPCC7002_A1836 deletion strain did not show many differences compared to the WT when the two strains were grown under standard conditions (**Figure 2C**). In fact, excluding some prophage genes (see below), strongly increased transcript levels were only observed for two genes: SYNPCC7002_A0792 and SYNPCC7002_A0793. BLAST searches and searches for conserved domains revealed that SYNPCC7002_A0792 encodes thioredoxin reductase (*trxB*) and that SYNPCC7002_A0793 encodes peroxiredoxin. The products of these two genes form an electron transfer chain in which peroxiredoxin inactivates H_2_O_2_ by reduction (Lu and Holmgren, 2014). Oxidized peroxiredoxin receives electrons from reduced thioredoxin, which in turn receives electrons from the cellular NADPH pool via thioredoxin reductase. Interestingly, the transcript levels of thioredoxin genes, however, were not more abundant in the mutant strain compared to the WT. A gene cluster comprising several prophage genes also showed increased transcript abundance in the SYNPCC7002_A1836 mutant strain. However, we did not observe cell lysis that would indicate activation of a lytic phage in our experiments with *Synechococcus* 7002. In WT cells, the mRNA levels of those genes (SYNPCC7002_A1876 to SYNPCC7002_A1888) were very low, and the transcript levels were still rather low even in the mutant strain. The significance of these latter changes is not clear at this time.

Fe-limitation of a culture of the SYNPCC7002_A1836 deletion strain had a very large effect on the relative transcript abundance (RTA) (**Figure 2D**). Many genes had strongly increased mRNA levels compared to a culture that was grown under standard conditions. Those genes (e.g., *isiA, isiB, sufA, sufB, sufD*, SYNPCC7002_G0018-G0025, SYNPCC7002_G0079-G0082) have been previously reported to be induced upon Fe-limitation in the WT (Ludwig and Bryant, 2012a), and these genes are not further induced upon Fe-limitation in the SYNPCC7002_A1649 merodiploid strain because they are already de-repressed to the maximum in the knock-down strain. Another set of genes showed slightly lower transcript levels in Fe-limited cells of the SYNPCC7002_A1836 mutant (e.g., *cpcA, cpcB, cpcC, apcA, apcB*). These genes generally have lower transcript levels under any nutrient limitation (Ludwig and Bryant, 2012a), and their transcript levels were also lower in the SYNPCC7002_A1649 merodiploid strain. To summarize, the transcriptome profile of iron-limited SYNPCC7002_A1836 mutant was essentially the same as iron-starved WT.

The transcriptome analysis of the third deletion mutant (SYNPCC7002_A2498) also showed increased mRNA levels of some genes compared to the WT when both strains were cultivated under standard conditions (**Figure 2E**). Those genes are basically located in two clusters. The first cluster is located on the chromosome adjacent to the SYNPCC7002_A2498 regulator (SYNPCC7002_A2499-SYNPCC7002_A2500-SYNPCC7002_A2501 (*zntC*)), and these genes are predicted to encode the components of an ABC-transporter for zinc. The second cluster, composed of *hemB-II, folE-II*, SYNPCC7002_G0131 and SYNPCC7002_G0132 is located on plasmid pAQ7. These genes encode a delta-aminolevulinic acid dehydratase paralog, a GTP cyclohydrolase I, a conserved hypothetical protein (BLAST and conserved domain searches showed that it harbors a methylmalonyl-coenzyme A mutase domain and has similarities to GTPases), and an FAD-dependent oxidoreductase, respectively. Interestingly, two genes located within this cluster, SYNPCC7002_G0129 and SYNPCC7002_G0130, did not show higher transcript levels in the SYNPCC7002_A2498 mutant strain. SYNPCC7002_G0129 is annotated as phosphoesterase, and it has homologs in other cyanobacterial strains, whereas SYNPCC7002_G0130 does not show conserved domains and no homologs were found in other organisms. In addition to these two clusters there is a single gene, *smtA*, which is more highly expressed in the SYNPCC7002_A2498 deletion strain compared to the WT. The *smtA* gene encodes a metallothionein, which is a cysteine-rich, heavy metal-binding protein (Ruttkay-Nedecky et al., 2013).

The transcriptome of the SYNPCC7002_A2498 deletion mutant was also probed under iron limitation and compared to that of a culture grown under standard conditions (**Figure 2F**). This comparison closely resembles that obtained for the SYNPCC7002_A1836 deletion strain and that of the WT when comparing Fe-limitation to standard growth (see above).

### Accumulation of Reactive Oxygen Species in *fur* Mutant Strains

**FIGURE 3:**
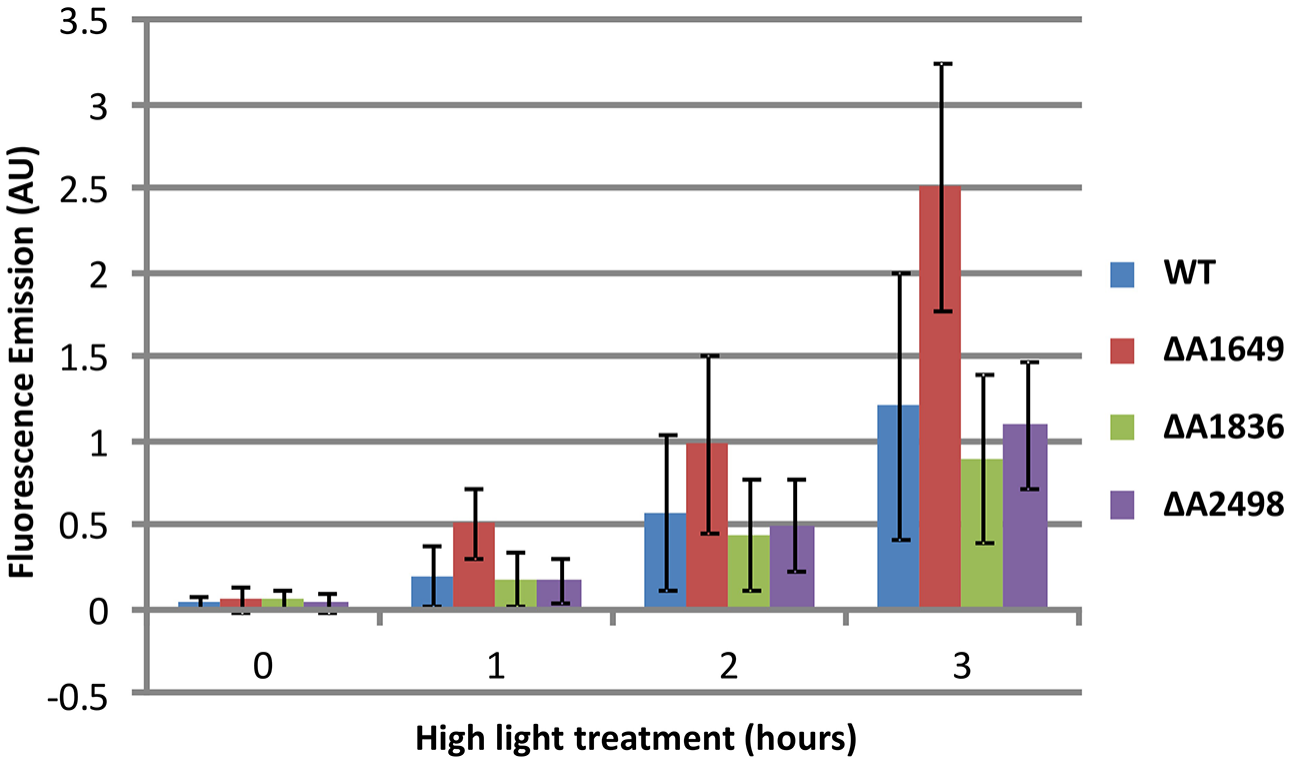
**Accumulation of ROS/RNS as detected by CM-H2DCFDA in cultures of *Synechococcus* 7002 wild type and SYNPCC7002_A1649 (*fur*), SYNPCC7002_A1836 and SYNPCC7002_A2498 mutant strains exposed to high light.** Cultures of wild type *Synechococcus* 7002 and SYNPCC7002_A1649 (*fur*), SYNPCC7002_A1836, and SYNPCC7002_A2498 mutant strains were grown to OD_730 nm_ = 0.7 under standard conditions, and then CM-H2DCFDA was added and the cultures were exposed to high light (830 μmol photons m^-2^ s^-1^). Samples were taken from two independent cultures and measured in duplicate after 0 h, 1 h, 2 h, and 3 h of high light incubation. The cultures were adjusted to OD_730 nm_ = 0.5 for subsequent fluorescence emission measurements.

To investigate further the function of the single Fur-type regulators, the relative ability of each strain to prevent formation and accumulation of ROS/RNS was compared to the WT. Cultures of *Synechococcus* 7002, which are known to be tolerant to very high light intensities (Sakamoto and Bryant, 2002; Nomura et al., 2006; Bernstein et al., 2014), were exposed to a high irradiance (830 μmol photons m^-2^ s^-1^) after growth under standard conditions. This treatment results in the enhanced production of ROS/RNS species, which can be detected by a fluorescent dye as described in the Section “Materials and Methods” (also see Eruslanov and Kusmartsev, 2010; Zhu et al., 2010). The dye reacts with ROS/RNS to form a fluorescent compound, and over the course of 3 h of high irradiance treatment an increase of ROS/RNS was observed for the WT (**Figure 3**). The SYNPCC7002_A1836 and SYNPCC7002_A2498 deletion mutants accumulated ROS/RNS at about the same levels as the WT, whereas the merodiploid SYNPCC7002_A1649 strain displayed a strong increase of ROS/RNS accumulation compared to the WT and the other Fur-type regulator mutants. The SYNPCC7002_A1836 mutant appeared to have slightly lower ROS/RNS levels than the WT.

### Zinc Tolerance and Effect of Zinc Limitation in *Synechococcus* 7002

To investigate the effect of zinc on *Synechococcus* 7002 cells, cultures were subjected to increased zinc levels and attempts were also made to induce zinc limitation. The standard A^+^ growth medium used for this study contains 2.3 μM Zn^2+^. Additional ZnCl_2_ (10 μM, 20 μM, 50 μM, 100 μM, and 200 μM), was added to cultures of the WT and the SYNPCC7002_A2498 mutant, and cultures were grown under otherwise standard conditions. Both the WT and the SYNPCC7002_A2498 mutant failed to grow at a Zn^2+^ concentration of 200 μM. At Zn^2+^ concentrations of 102 μM and below, both WT and mutant grew equally well, and even at 102 μM Zn^2+^ the WT *Synechococcus* 7002 showed the same growth behavior compared to growth in normal A^+^ medium (data not shown). Attempts to subject *Synechococcus* 7002 cultures to zinc limitation were performed in zinc-free A^+^ medium and by adding TPEN as a chelator for divalent cations. TPEN as a chelator, however, has an even higher affinity to the divalent forms of nickel, copper, cobalt and cadmium, and Fe^2+^ is also complexed by TPEN, though with lower affinity (Anderegg et al., 1977; Sigdel et al., 2006). However, no difference in growth of the WT and the SYNPCC7002_A2498 mutant was observed, even after several transfers.

**FIGURE 4:**
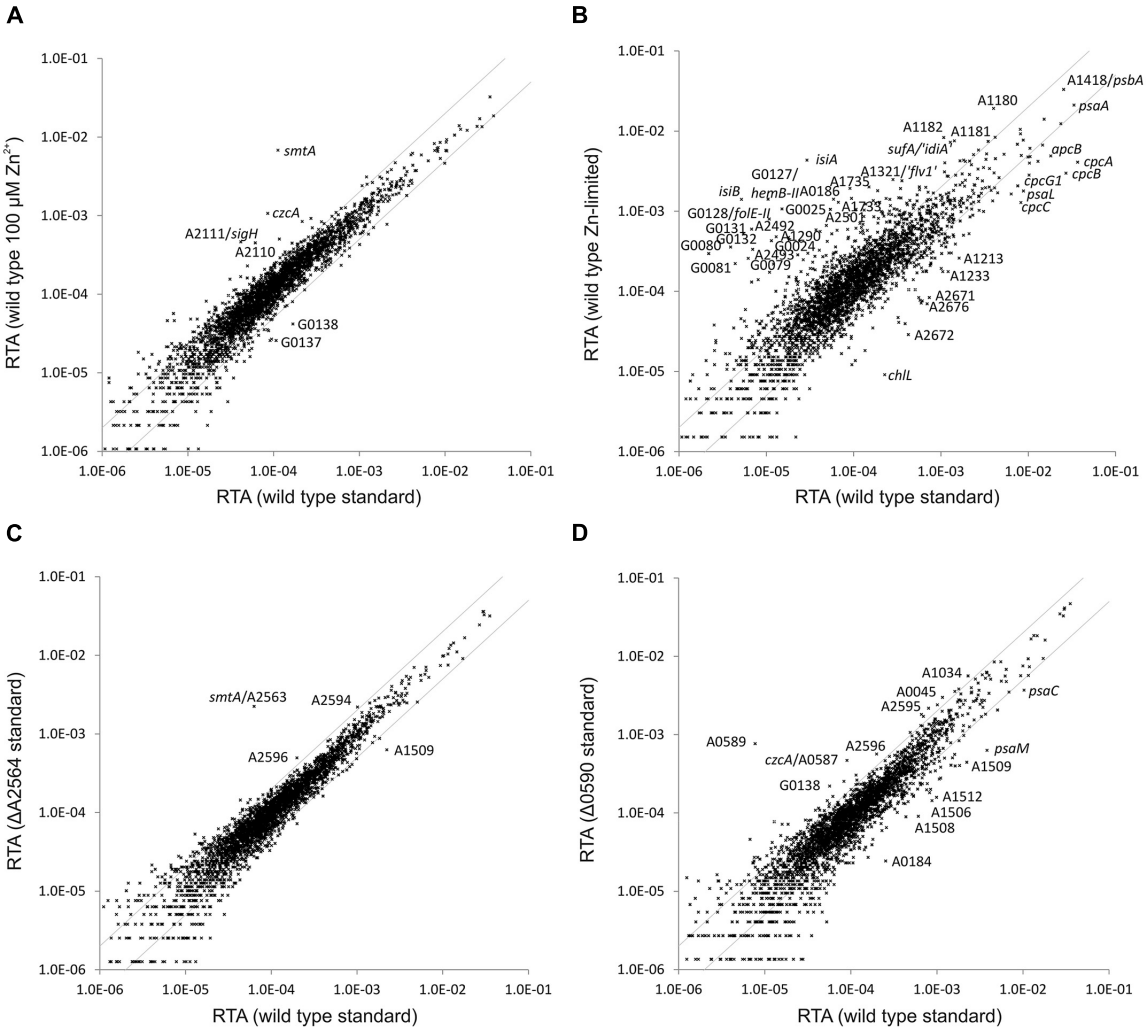
**Relative transcript abundances for *Synechococcus* 7002 wild type cultures grown at altered zinc levels compared to standard growth conditions and of the SYNPCC7002_A2564 (*smtB*) and SYNPCC7002_A0590 deletion mutants compared to the *Synechococcus* 7002 wild type.** The scatter plots show **(A)** the RTAs of a wild type *Synechococcus* 7002 culture grown in presence of high zinc (102 μM) compared to a culture grown under standard conditions; **(B)** the RTAs of a culture of wild type *Synechococcus* 7002 culture grown under zinc limitation compared to a culture grown under standard conditions; **(C)** RTAs of the SYNPCC7002_A2564 (*smtB*) deletion mutant compared to the wild type, both grown under standard conditions; **(D)** RTAs of the SYNPCC7002_A0590 deletion mutant compared to a wild type culture, both grown under standard conditions. The values for the wild type under standard conditions were calculated as the mean of three independent biological replicates. The gray lines show twofold changes in either direction. Selected genes are identified by gene locus or abbreviated locus tag number. For details, see text.

*Synechococcus* 7002 cultures grown at high Zn^2+^ concentration (100 μM) and a culture grown in the presence of 50 μM TPEN were subjected to transcriptome profiling. Very few genes showed a variation of the transcript level in the culture grown at a high zinc level compared to a culture grown in A^+^ medium under standard conditions (**Figure 4A**): *smtA* (encoding a metallothionein, see above), *czcA* (annotated as cation eﬄux system protein CzcA) and, interestingly, *sigH* (sigma factor H; SYNPCC7002_A2111) and the adjacent ORF SYNPCC7002_A2110 (hypothetical protein; not well conserved according to BLAST searches) had higher transcript levels. The *sigH* gene and ORF SYNPCC7002_A2110 have been reported to be co-transcribed, but the function of the latter remained obscure (Inoue-Sakamoto et al., 2007). SYNPCC7002_G0137 (annotated as MotA/TolQ/ExbB proton channel family protein, a biopolymer transport protein) and SYNPCC7002_G0138 (annotated as TonB-dependent siderophore receptor) showed lower transcript levels.

The RTA pattern of a culture grown in the presence of 50 μM TPEN (to induce zinc limitation) showed more differences relative to a culture grown in A^+^ medium under standard conditions (**Figure 4B**). The pattern resembles the one obtained for iron-limited cultures: several genes related to iron uptake and low iron adaptation (e.g., *isiA, isiB*, SYNPCC7002_G0079–SYNPCC7002_G0081) were induced and genes encoding components of the light harvesting complex (*apcB, cpcA, cpcB, cpcC*) displayed a lower mRNA level. In addition there are two sets of genes *hemB-II* (delta-aminolevulinic acid dehydratase), *folE-II* (GTP cyclohydrolase), SYNPCC7002_G0131–SYNPCC7002_G0132 (a conserved hypothetical protein and a FAD-dependent oxidoreductase, respectively) located on plasmid pAQ7 and SYNPCC7002_A2499–SYNPCC7002_A2501 (encoding components of a zinc-ABC transporter) located on the chromosome having a sharply induced mRNA level upon TPEN addition. These two clusters also showed a higher expression level in the SYNPCC7002_A2498 deletion mutant strain compared to the WT (see above).

### Effect of *smtB* Deletion on the Global Transcriptome

In presence of a high Zn^2+^ level and in the SYNPCC7002_A2498 deletion mutant strain (in A^+^ medium under standard conditions), transcript levels for *smtA* (encoding metallothionein; SYNPCC7002_A2563) increased dramatically. A gene annotated as metallothionein repressor (*smtB*; SYNPCC7002_A2564) occurs adjacent to *smtA*, an organization that exists for an orthologous regulator in other cyanobacterial strains, either in combination with the gene encoding metallothionein or with a zinc exporter (Morby et al., 1993; Thelwell et al., 1998). In order to investigate the regulon of this transcriptional repressor in *Synechococcus* 7002, the *smtB* gene was deleted. The WT and mutant alleles for the *smtB* deletion mutant segregated fully in *Synechococcus* 7002, and the resulting mutant strain grew similarly to the WT (data not shown). The *smtB* deletion mutant strain was subjected to transcriptome profiling under standard growth conditions, and the resulting transcriptome was compared to that of the WT (**Figure 4C**). Only a single gene (*smtA*) had a much higher mRNA level in the *smtB* deletion strain compared to the WT. Transcript levels of essentially all other genes with relatively high expression levels changed less than twofold compared to the WT. Transcript levels for SYNPCC7002_A2594 and SYNPCC7002_A2596 (both hypothetical proteins) were slightly above the twofold higher level, and transcript levels for SYNPCC7002_A1509 (annotated as putative membrane protein) were about 3.5-fold lower compared to the WT.

### Effect of SYNPCC7002_A0590 Deletion on the Global Transcriptome

The *czcA* gene (annotated as cation eﬄux system protein CzcA) showed the second highest increase of the mRNA level in cells of WT *Synechococcus* 7002 in the presence of high Zn^2+^. The *czcA* gene is located within a gene cluster (SYNPCC7002_A0585 –SYNPCC7002_A0591) that also harbors a gene annotated as arsenical resistance operon repressor, ArsR family (SYNPCC7002_A0590). To characterize the regulon of SYNPCC7002_A0590, a deletion mutant was constructed for this gene. Mutant and WT alleles fully segregated in the transformants, showing that the product of SYNPCC7002_A0590 is not required for viability under standard growth conditions. Consistent with this, the mutant strain grew similarly to the WT in A^+^ medium under standard conditions (data not shown). The transcriptome of the SYNPCC7002_A0590 mutant strain was determined for a culture grown under standard conditions and compared to that of the WT (**Figure 4D**). Two genes within the cluster (SYNPCC7002_A0589, annotated as arsenite eﬄux pump ACR3, and SYNPCC7002_A0588 annotated as low molecular weight phosphotyrosine protein phosphatase), which are located just downstream of the regulator SYNPCC7002_A0590, showed a much higher expression level in the SYNPCC7002_A0590 deletion mutant compared to the WT (about 150- and 100-fold higher, respectively). The next gene within the cluster, *czcA*, had an about fivefold higher mRNA level in the mutant strain. Other genes located in the same gene cluster displayed only minor changes in the transcript levels in the deletion mutant relative to the WT: these are SYNPCC7002_A0591 (annotated as eﬄux transporter, RND family, MFP subunit), which is located upstream of SYNPCC7002_A0590, SYNPCC7002_A0586 (annotated as probable dioxygenase of extradiol dioxygenase family) and SYNPCC7002_A0585 (annotated as outer membrane eﬄux protein), both located downstream from *czcA*. A few other genes with relatively high transcript levels showed slightly higher transcript levels in the SYNPCC7002_A0590 deletion mutant compared to the WT; among those were SYNPCC7002_A2595 and SYNPCC7002_A2596, both annotated as conserved hypothetical proteins, which exhibited a ∼3-fold increase in transcript levels. Interestingly, those genes displayed slightly higher transcript levels in the *smtB* mutant. An elevated zinc level (100 μM zinc in the culture or SYNPCC7002_A2498 deletion), however, left the transcript level of those genes unaffected, whereas zinc limitation induced by TPEN caused ∼1.5-fold and ∼4-fold lower expression levels for SYNPCC7002_A2595 and SYNPCC7002_A2596, respectively. Among those genes that had a relatively high transcript abundance and that showed somewhat lower transcript levels in the SYNPCC7002_A0590 mutant strain relative to the WT, were SYNPCC7002_A1506 to SYNPCC7002_A1512, which showed up to ∼7-fold lower expression levels; these genes included glycosyl transferases, a pyruvyltransferase, and a sulfotransferase.

## Discussion

**FIGURE 5:**
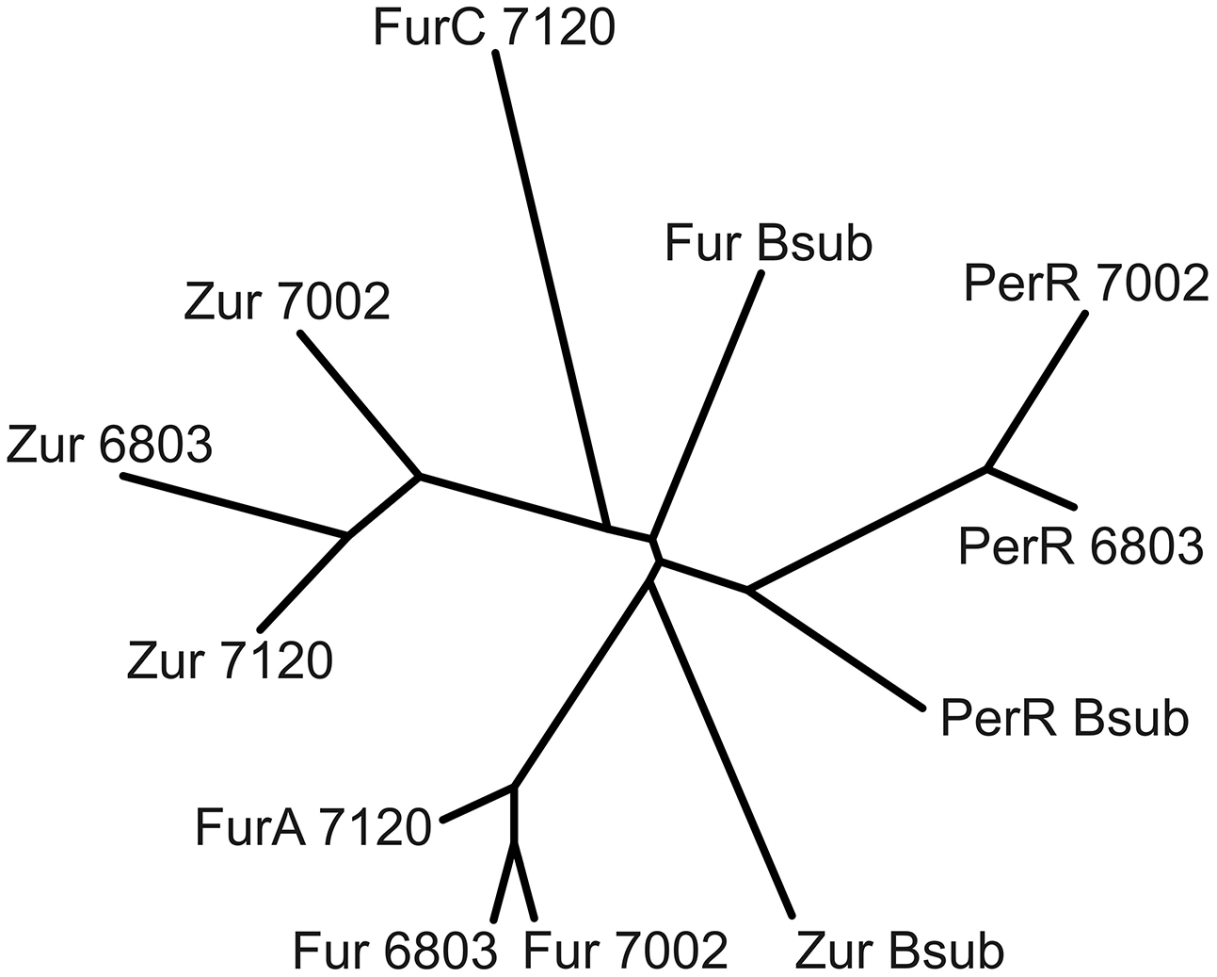
**Phylogenetic tree of Fur-type regulators from three cyanobacterial strains.** Amino acid sequences of the three Fur-type regulators from *Anabaena* 7120, *Synechocystis* 6803, and *Synechococcus* 7002 were aligned using ClustalW and a phylogenetic tree was generated using (Protein alignment, BLOSUM matrix, default settings; unrooted neighbor-joining tree with branch lengths). The three respective Fur-type regulators (Fur, Zur, and PerR) from *Bacillus subtilis* were used as an outgroup.

Fur-type regulators are present in many cyanobacterial strains, and target genes and functions have been assigned to specific regulators in a few cyanobacterial strains (Ghassemian and Straus, 1996; Kobayashi et al., 2004; Li et al., 2004; López-Gomollón et al., 2009; González et al., 2010, 2011, 2014; Napolitano et al., 2012). However, no previous study has investigated all of the Fur-type regulators in a single cyanobacterium using the same experimental conditions. A phylogenetic comparison of the three Fur-type regulators of *Synechococcus* 7002 with those of *Synechocystis* sp. PCC 6803, *Anabaena* sp. PCC 7120 and *Bacillus subtilis* shows that SYNPCC7002_A1649 clusters with *Anabaena* 7120 FurA and Fur from *Synechocystis* 6803 (**Figure 5**). SYNPCC7002_A2498 clusters with Zur from *Anabaena* 7120 and from *Synechocystis* 6803; however, the similarity is not that high compared to the first cluster comprising of Fur/FurA. The third Fur-type regulator, SYNPCC7002_A1836, is similar to PerR from *Synechocystis* 7002. Interestingly, the third Fur-type regulator (FurC) from *Anabaena* 7120 does not fall into this cluster and is distinct from the cyanobacterial clusters. As expected, the Fur-type regulators from *B. subtilis*, which were used as outgroups, are somewhat different from the respective cyanobacterial clusters.

In this study the targets for these three Fur-type regulators were investigated in *Synechococcus* 7002, and to our knowledge this is the first transcriptome profiling study on a merodiploid knock-down strain of an iron-responsive Fur-type regulator gene. The transcriptome profiling results clearly show that all three Fur-type regulators are transcriptional repressors and that one (SYNPCC7002_A1649, annotated as *fur*) is an important regulator of iron homeostasis. Transcript levels of genes, which are specifically responsive to iron limitation (Ludwig and Bryant, 2012a), were maximally de-repressed in the merodiploid SYNPCC7002_A1649 strain, and expression levels of genes that respond to any other type of nutrient limitation were unchanged as long as no iron limitation was induced. Similar to the results obtained in this study, previous studies of the *fur* gene in other cyanobacteria reported that it was not possible to delete this gene, indicating that Fur is required for cell viability (Ghassemian and Straus, 1996; González et al., 2011). Studies in *Anabaena* 7120 based on *in silico* predictions, promoter binding experiments and proteomic studies on over-expression of FurA in its natural host revealed a number of target genes in that cyanobacterial strain (González et al., 2011, 2014). Interestingly, besides iron homeostasis those target genes in *Anabaena* 7120 belonged to additional functional categories, e.g., photosynthesis, respiration, heterocyst differentiation, oxidative stress response and light-dependent signal transduction mechanisms. These findings are clearly different from the results obtained here for a *fur* knock-down strain of *Synechococcus* 7002, for which the Fur regulon seems to be restricted to the principal function, iron homeostasis. The fact that we could achieve a maximal de-repression by having just a 2.5-fold lower transcript level of the regulator was surprising. Assuming that the mRNA quantity approximately translates to the protein level, this would mean that the cellular level or Fur protein is below the threshold that is required for a full transcriptional repression and that even excess iron as co-repressor could not compensate for that. A low level of the co-repressor iron – as induced in the iron limitation experiment – did not result in significantly higher de-repression, suggesting that only low levels of the functional repressor can be present in the merodiploid SYNPCC7002_A1649 strain.

Discounting the viral genes of unknown function, the second Fur-type regulator, SYNPCC7002_A1836, has only two target genes in *Synechococcus* 7002, which were de-repressed in the deletion mutant. Those two genes encode peroxiredoxin and thioredoxin reductase, both of which involved in detoxification of ROS. A study of a homologous gene, named *perR* in *Synechocystis* 6803, produced similar results; however, microarray analysis indicated that the number of genes in the PerR regulon of *Synechocystis* 6803 was much higher, and they were functionally more diverse (Li et al., 2004). On the other hand, a global transcriptome study of oxidative stress induced by treatment with methyl viologen in *Synechococcus* 7002 (Ludwig and Bryant, 2012b), also showed a very limited response to oxidative stress.

Deletion of the third Fur-type regulator gene (SYNPCC7002_A2498) resulted in de-repression of genes encoding zinc-transporters and enzymes that are paralogs of zinc-containing enzymes encoded elsewhere in the genome. The regulon of the zinc-uptake regulator (Zur) has been well studied in *Anabaena* 7120 (Napolitano et al., 2012). In *Anabaena* 7120 a total of 23 genes, organized in six operons and six single transcriptional units, are targets of Zur, whereas in *Synechococcus* 7002 the Zur regulon comprises only two clusters with a total of seven target genes. Similar to the target genes of the other two Fur-type regulators, the functional diversity of Zur targets seems to be more limited in *Synechococcus* 7002. Functional assignments of the target genes in *Synechococcus* 7002 included only a subset of those reported for *Anabaena* 7120 (Napolitano et al., 2012).

Based on the similarities of the three Fur-type regulators from *Synechococcus* 7002 to those of *Anabaena* 7120 and *Synechocystis* 6803 on the amino acid sequence level and on the functional level (target genes), we propose the following names for the three genes encoding Fur-type regulators in *Synechococcus* 7002: SYNPCC7002_A1649 is *fur*, SYNPCC7002_A1836 *perR*, and SYNPCC7002_A2498 is named *zur*.

Metal homeostasis and oxidative stress are intimately related, and thus it was not surprising that oxidative stress was exacerbated in some of the mutants produced in this study. In particular, inappropriate expression of the Fur regulon strongly exacerbated oxidative stress induced by high light. ROS/RNS levels in cells accumulated much higher in the *fur* merodiploid strain than in the WT after a 3-h exposure to high irradiance. This is probably due to increased iron uptake, resulting in a higher cellular Fe level, which may lead to increased ROS formation via the Fenton reaction (Kozlowski et al., 2014). This observation provides a likely explanation for why the *fur* gene cannot be fully deleted: the resulting intracellular iron levels, and thus the resulting ROS/RNS levels, might exceed the cellular capacity to inactivate ROS/RNS—even for an extremely stress tolerant strain like *Synechococcus* 7002. Inappropriate expression of the Zur regulon did not enhance ROS/RNS production in response to high irradiance. Derepression of peroxiredoxin and thioredoxin reductase in the mutant lacking PerR had at most a very small effect in reducing ROS/RNS.

*Synechococcus* 7002 cultures can grow in the presence of Zn^2+^ levels as high as 100 μM, and growth was almost unaffected up to this level. However, absolutely no growth was observed at 200 μM Zn^2+^. Interestingly, the *zur* mutant strain grew equally well compared to the WT, although transcriptome data indicated an increased expression of the zinc regulon. This probably means that detoxification mechanisms for intracellular zinc/heavy metals, metallothionein (SmtA) and the cation eﬄux system protein CzcA, are still able to balance the increased zinc import to maintain homeostasis. Zinc limitation could be induced in *Synechococcus* 7002 cultures by adding the chelator TPEN, resulting in an increase of transcript levels of genes under control of Zur. This is consistent with data obtained for zinc limitation in *Anabaena* 7120 (Napolitano et al., 2012). However, using TPEN as a chelator for divalent cations for *Synechococcus* 7002 also clearly resulted in iron limitation as indicated by increased transcript levels of genes related to iron uptake and the Fur regulon. This result is very similar to observations made in a transcriptome profiling study on metal-limitation in *Escherichia coli* (Sigdel et al., 2006). The lack of specificity of TPEN makes it difficult to prepare and study cells that are solely zinc-limited.

In addition to studying the regulons of the three Fur-type regulators, we investigated the targets of two other transcriptional repressors of the ArsR-SmtB family that are related to a heavy metal stress response. The SmtB transcriptional repressor of *Synechococcus* 7002 has only one target gene, *smtA*, encoding metallothionein. This regulation pattern is the same as for *Synechococcus* sp. PCC 7942 (Morby et al., 1993); however, in that study additional targets cannot be excluded since no global approach was performed. *Synechocystis* 6803 also encodes *smtB*, but in this cyanobacterium a zinc exporter, *ziaA*, is the target gene rather than *smtA* (Thelwell et al., 1998), also this study did not include a global screen for other potential targets.

The transcriptional regulator SYNPCC7002_A0590, annotated as an arsenical resistance repressor, ArsR, is a repressor for four genes within a cluster adjacent to SYNPCC7002_A0590 in *Synechococcus* 7002. The highest de-repression was observed for the two genes just downstream of the regulator, one of which encodes an arsenite eﬄux pump ACR3. One of the next two genes further downstream, which showed a lower level of de-repression, encodes the cation eﬄux system protein CzcA. This is an interesting observation, because SYNPCC7002_A0590 seems to regulate multiple functions, at least according to its target genes. The ArsR-SmtB family of transcriptional repressors displays a broad variety both with respect to the metal ions, which de-repress these regulators, and to the target genes (Busenlehner et al., 2003; Osman and Cavet, 2010). The main function for SYNPCC7002_A0590 seems to be the regulation of arsenite eﬄux as indicated by the high level of de-repression of the gene encoding an arsenite eﬄux pump. However, in *Synechococcus* 7002 this ArsR-type transcriptional repressor has additional target genes, although they are de-repressed to a lesser extent. Finally, the target genes of both SmtB and SYNPCC7002_A0590, *smtA* and *czcA*, respectively, are derepressed in response to elevated zinc level identifying zinc at least as one de-repressor of both transcriptional regulators. Therefore, together with Zur, these two ArsR-SmtB family regulators form a regulatory network that controls zinc homeostasis in *Synechococcus* 7002.

## Conclusion

In this study we demonstrated the target genes of the three Fur-type regulators Fur, PerR and Zur of *Synechococcus* 7002, which were fewer in number compared to the respective regulons that have been reported for other cyanobacterial strains through microarray studies. Furthermore, we presented the target genes for two additional transcriptional repressors that are involved in zinc homeostasis in addition to Zur. We suggest that the Fur transcription factor is essential for cyanobacterial viability because de-repression of the Fur regulon can exacerbate oxidative stress in response to high irradiance.

## Conflict of Interest Statement

The authors declare that the research was conducted in the absence of any commercial or financial relationships that could be construed as a potential conflict of interest.

## Abbreviations

CM-H_2_DCFDA: 5-(and-6)-chloromethyl-2′,7′-dichlorodihydrofluorescein diacetate acetyl ester
PCR: polymerase chain reaction
ROS/RNS: reactive oxygen/nitrogen species
sRNA: small regulatory RNA
TPEN: N,N,N’,N’-*tetrakis-*(2-pyridylmethyl)ethane-1,2-diamine
WT: wild type
